# Proteomic analysis of *Fasciola gigantica* excretory and secretory products (*Fg*ESPs) co-immunoprecipitated using a time course of infected buffalo sera

**DOI:** 10.3389/fmicb.2022.1089394

**Published:** 2022-12-23

**Authors:** Mengwei Zheng, Xuelian Jiang, Xinping Kong, Yanfeng Guo, Weiyu Zhang, Wenda Di

**Affiliations:** ^1^College of Animal Science and Technology, Guangxi University, Nanning, China; ^2^Guangxi Zhuang Autonomous Region Engineering Research Center of Veterinary Biologics, Nanning, China; ^3^Guangxi Key Laboratory of Animal Reproduction, Breeding and Disease Control, Nanning, China; ^4^Guangxi Colleges and Universities Key Laboratory of Prevention and Control for Animal Disease, Nanning, China

**Keywords:** *Fasciola gigantica*, co-immunoprecipitation, excretory and secretory products, interaction, LC–MS/MS, screening

## Abstract

**Introduction:**

Widespread *Fasciola gigantica* infection in buffaloes has caused great economic losses in buffalo farming. Studies on *F. gigantica* excretory and secretory products (*Fg*ESP) have highlighted their importance in *F. gigantica* parasitism and their potential in vaccine development. Identifying *Fg*ESP components involved in *F. gigantica*-buffalo interactions during different periods is important for developing effective strategies against fasciolosis.

**Methods:**

Buffaloes were assigned to non-infection (*n* = 3, as control group) and infection (*n* = 3) groups. The infection group was orally administrated 250 metacercariae. Sera were collected at 3, 10, and 16 weeks post-infection (wpi) for the non-infection group and at 0 (pre-infection), 1, 3, 6, 8, 10, 13, and 16 wpi for the infection group. *Fg*ESP components interacting with sera from the non-infection and infection groups assay were pulled down by co-IP and identified using LC–MS/MS. Interacting *Fg*ESP components in infection group were subjected to Kyoto Encyclopedia of Genes and Genomes (KEGG) metabolic pathway and gene ontology (GO) functional annotation to infer their potential functions.

**Results and discussion:**

Proteins of *Fg*ESP components identified in the non-infection group at 3, 10, and 16 wpi accounted for 80.5%, 84.3%, and 82.1% of all proteins identified in these three time points, respectively, indicating surroundings did not affect buffalo immune response during maintenance. Four hundred and ninety proteins were identified in the infection group, of which 87 were consistently identified at 7 time points. Following GO analysis showed that most of these 87 proteins were in biological processes, while KEGG analysis showed they mainly functioned in metabolism and cellular processing, some of which were thought to functions throughout the infection process. The numbers of specific interactors identified for each week were 1 (*n* = 12), 3 (*n* = 5), 6 (*n* = 8), 8 (*n* = 15), 10 (*n* = 23), 13 (*n* = 22), and 16 (*n* = 14) wpi, some of which were thought to functions in specific infection process. This study screened the antigenic targets in *Fg*ESP during a dense time course over a long period. These findings may enhance the understanding of molecular *F. gigantica*-buffalo interactions and help identify new potential vaccine and drug target candidates.

## 1. Introduction

Fasciolosis is a widespread zoonotic disease caused by *Fasciola hepatica* and *Fasciola gigantica* that primarily affects public health and economically important livestock. It is considered as one of the top 17 neglected tropical diseases (Piedrafita et al., 2010). *Fasciola hepatica* mainly infects sheep and cattle worldwide, while *F. gigantica* mainly infects buffalo in the subtropic and tropic zones (Doy and Hughes, 1984; Chen et al., 2000; Zhang et al., 2005; Aghayan et al., 2019; Niedziela et al., 2021). Since its infection of livestock leads to annual economic losses of >$3 billion worldwide (Calvani and Šlapeta, 2021). In addition, at least 2.4 million individuals are infected worldwide and 180 million are at risk of new infections (Meemon and Sobhon, 2015). Despite affecting human and livestock health in an area that represents up to 77% of the global population, research interest in *F. gigantica* consistently lags behind that of *F. hepatica* (Agatsuma et al., 2000), and little is known about the factors that contribute to the pathogenicity and virulence of *F. gigantica*.

*F. gigantica* metacercariae ingestion by the definitive host leads to excystation and the release of newly excysted juveniles (NEJs) that burrow through the duodenal wall into the peritoneum. They then move toward the liver and penetrate the liver capsule. The immature flukes migrate through the liver for 11 weeks, reaching and maturing in the bile ducts for 12–16 weeks post-infection (wpi), after which they commence egg laying (Calvani and Šlapeta, 2021). The *F. gigantica* life cycle in definitive mammalian hosts largely relies on excretory and secretory products (*Fg*ESP) since they act as antigens that stimulate humoral and cell-mediated immunity and also function in fluke survival and host–parasite interactions (El-Ghaysh et al., 1999; Zhang et al., 2006; Novobilsky et al., 2007; Hacariz et al., 2011; Wang et al., 2021). Some *FgESP* components, such as cathepsin L1, cathepsin B, saposin-like protein 2 (SAP-2), have been identified to identify potential vaccine candidates (Chantree et al., 2013; Kueakhai et al., 2013, 2015).

Previous proteomic studies have shown that the *Fg*ESP release profile varies across three developmental stages: the NEJ 24 h post-excystment, immature fluke 21 days post-infection (immature), and adult (Lalor et al., 2021). In the early infection stage, NEJs secrete a range of stage-specific peptidases and proteolytic-related proteins to break down extracellular matrix components that maintain tissue integrity and participate in fluke invasion (Di Maggio et al., 2019; Davey et al., 2022). During the liver migratory phase, immature fluke secretions are dominated by peptidases involved in blood digestion, cathepsin peptidases, and their inhibitors to support tissue penetration and blood feeding (Lalor et al., 2021). Once adults arrive at the bile duct, they feed on and detoxify bile components by expressing cathepsin L and B peptidases, enzymes, peptidase inhibitors, legumain, helminth defense molecules, and glycoproteins (Meemon et al., 2004; Ghosh et al., 2005; Adisakwattana et al., 2007; Sansri et al., 2013; Ryan et al., 2020; Cwiklinski and Dalton, 2022), some of which function in immunoregulation (Ticho et al., 2020). Therefore, it is vital to identify the *Fg*ESP components produced by *F. gigantica* at different developmental stages to understand molecular buffalo-*F. gigantica* interactions and the *F. gigantica* development process in buffalo. However, difficulties in obtaining parasites at different developmental stages *in vivo* and *in vitro* make it impossible to obtain and study *Fg*ESPs at different developmental stages. Consequently, *Fg*ESPs produced by adults, which can be easily obtained, were identified in buffalo serum during different *F. gigantica* infection periods to identify changes in them.

Huang first explored the interaction of *FgESP* with buffalo serum at three-time points (6, 10, and 14 wpi; Huang et al., 2019a). Considering the complex interaction mechanisms between *F. gigantica* and buffalo at larval and adult stages, it is still required to conduct continuous and periodic observations concerning host-pathogen interactions. Furthermore, the recent completion of genome and transcriptome sequencings (Zhang et al., 2017; Pandey et al., 2020; Luo et al., 2021) enables us to obtain more protein sequence information (UniProt *F. gigantica* database; downloaded on 2021/11/19) of *F. gigantica* in public databases. The sera of *F. gigantica*-infected buffalo were collected at seven time points (1, 3, 6, 8, 10, 13, and 16 wpi) and co-immunoprecipitated (co-IP) to pull down *Fg*ESP components that interacted with them. These components were characterized by liquid chromatography–tandem mass spectrometry (LC–MS/MS) and bioinformatics. This approach can be used to analyze specific proteins and provide a reliable basis for the screening of diagnostic antigens of *F. gigantica*.

## 2. Materials and methods

### 2.1. Preparation of buffalo serum representing different infection periods

*Fasciola gigantica* metacercariae were collected from *Galba pervia* experimentally infected with miracidia, encysted on 4 cm^2^ polythene strips, and stored in distilled water at 4°C until required. Each metacercariae batch was examined for viability and then counted.

Six 6-month-old buffaloes of Murrah, Nili-Ravi, Mediterranean, and their crossbreds with indigenous buffaloes in Guangxi (China) were randomly assigned to non-infection (A1, A2, and A3) and infection (B1, B2, and B3) groups, with three in each group (Supplementary Table S1). They were stall-fed on a balanced diet in the dairy of the Buffalo Research Institute, Chinese Academy of Agricultural Sciences, and Guangxi Zhuang Nationality Autonomous Region. They were confirmed free from fluke infection through indirect *Fg*ESP enzyme-linked immunosorbent assays (ELISA; Supplementary Table S2) and coprological examination (Zhang et al., 2006). In week 0, the infection group was given a gelatine capsule containing 250 viable *F. gigantica* metacercariae, while the non-infection group were mock-inoculated with 0.85% sodium chloride solution without metacercariae, the mean numbers of flukes recovered were 55.5 ± 14.1 (22.2 ± 5.6 of infection dose) in infection group (Wang et al., 2022b). Whole blood was collected from the non-infection (3, 10, and 16 wpi) and infection (0, 1, 3, 6, 8, 10, 13, and 16 wpi) groups for serum preparation and stored at −80°C until needed.

### 2.2. *Fg*ESP preparation

*Fg*ESPs were prepared as previously described (Novobilský et al., 2007). Briefly, adult *F. gigantica* were collected from infected buffaloes’ livers and washed three times in warm phosphate-buffered saline (PBS, pH 7.2) to remove the residual material. Next, flukes were incubated in sterile Roswell Park Memorial Institute (RPMI) 1,640 media supplemented with antibiotics and antimycotics (10,000 UI/ml penicillin G and 10 mg/ml amphotericin B) at 37°C for 2 h. Then, flukes were transferred into sterile RPMI 1640 media and incubated at 37°C for a further 5 h. After incubation, the supernatant was centrifuged at 2,500 *g* for 30 min at 4°C and then filtered through a 0.22 μm nylon filter. Finally, the supernatant was concentrated, freeze-dried into a powder, and stored at −80°C. Before use, the powder was dissolved in deionized water. Its protein concentration was determined using a Bicinchoninic Acid (BCA) Assay Kit (Beijing Solarbio Science & Technology Co., Ltd., China).

### 2.3. Co-IP of *Fg*ESP-antibody binding proteins

The Protein A/G Plus-Agarose Immunoprecipitation Kit (Santa Cruz Biotechnology, USA) was used to pull down the *Fg*ESP-serum antibody binding proteins according to the manufacturer’s instructions. For the non-infection group, 5 mg of *Fg*ESPs was incubated with 1 ml of serum (A1, A2, and A3 at 3, 10, and 16 wpi) and 20 μl of Protein A/G Plus-Agarose Beads at 4°C for 2 h. Next, pellets were collected by centrifugation at 1,000 *g* and 4°C for 5 min. Then, the pellets were washed three times with 500 μl PBS and centrifugation at 1,000 *g* and 4°C for 5 min. After the final washing, the sediment was resuspended in 50 μl PBS, and 10 μl was used for sodium dodecyl sulfate-polyacrylamide gel electrophoresis (SDS-PAGE) analysis. The remaining 40 μl was used for LC–MS/MS identification.

For the infection group, 5 mg of *Fg*ESPs was precleared (negative serum and *FgESP* pull down non-specific interaction proteins through Co-IP) by incubation with 1 ml of negative (week 0) serum and 20 μl of Protein A/G Plus-Agarose Beads at 4°C for 2 h. After pelleting the beads by centrifugation at 1,000 *g* and 4°C for 5 min, the supernatant was transferred and divided equally into three fresh tubes. Next, 500 μl of corresponding buffalo sera (B1, B2, and B3 at 1, 3, 6, 8, 10, 13, and 16 wpi) was added to each tube with 20 μl of Protein A/G Plus-Agarose Beads and incubated at 4°C overnight. The pellet was collected by centrifugation at 1,000 *g* and 4°C for 5 min. The pellets were washed three times with 500 μl PBS and centrifugation at 1,000 *g* and 4°C for 5 min. After the final washing, sediments were resuspended in 50 μl PBS, and 10 μl was used for SDS-PAGE analysis. The remaining 40 μl was used for LC–MS/MS identification.

### 2.4. In-solution trypsin digestion

Liquid mass spectrometry (LMS) was performed by gel chromatography, and the protein solution was conducted to SDS-PAGE, then the targets band was extracted from the gel and cut into 0.5 mm cubes. Next, the decolorized gel was washed three times with acetonitrile solution until gelatinous particles were completely white. Then, 500 μl of 10 mM dithiothreitol was added and incubated at 56°C for 30 min. Next, 500 μl of a decolorizing solution was added and mixed at room temperature for 10 min. Then, the gelatinous particles were centrifuged at 3,000 *g* to remove the supernatant. Next, 500 μl of 55 mM iodoacetamide was added and incubated for a further 30 min at room temperature before being centrifuged at 3,000 *g*. Then, 500 μl of decolorizing solution was added and incubated for 10 min at room temperature before being centrifuged at 3,000 *g* to remove the supernatant. Next, 500 μl of acetonitrile was added until the micelles were completely whitened and then vacuum-dried for 5 min. Then, trypsin was added according to the gel volume and incubated in an ice bath for 30 min. Next, 25 mM ammonium bicarbonate (pH 8.0) was added and incubated at 37°C overnight. Then, 300 μl of extraction solution (60% acetonitrile and 5% formic acid) was added and sonicated for 10 min. Finally, the solution was centrifuged at 3,000 *g*, and the supernatant was collected and vacuum-dried.

### 2.5. LC–MS/MS analysis

The sample was dissolved with 20 μl of 0.2% trifluoroacetate, centrifuged at 10,000 rpm for 20 min, and dried with a vacuum concentrator (LaboGene, SCAN SPEED 40, Denmark). Samples were then adjusted to 1 μg/μL using the machine’s buffer. The sample volume was set to 5 μl, and the collection scan mode was set to 60 min. In the sample, we scanned for peptides with a mass-to-charge ratio of 350–1,200. The mass spectrometry data was collected using the Triple TOF 5600 + LC/MS system (AB SCIEX, USA). The peptide samples were dissolved in 2% acetonitrile with 0.1% formic acid and analyzed using the Triple TOF 5600 Plus mass spectrometer coupled with the Eksigent nanoLC system (AB SCIEX, USA). The peptide solution was added to the C18 capture (3 μm; 350 μm × 0.5 mm; AB Sciex, USA) and C18 analytical (3 μm; 75 μm × 150) columns with a 60 min time gradient and a 300 nl/min flow rate for gradient elution. The two mobile phases were buffers A (2% acetonitrile, 0.1% formic acid, and 98% water) and B (98% acetonitrile, 0.1% formic acid, 2% water). For information-dependent acquisition, the MS spectrum was scanned with a 250 ms ion accumulation time, and the MS spectrums of 30 precursor ions were acquired with a 50 ms ion accumulation time. The MS1 spectrum was collected in the range 350–1,200 m/z, and the MS2 spectrum was collected in the range 100–1,500 m/z. The precursor ion dynamic exclusion time was set to 15 s.

### 2.6. Data analysis

The raw MS/MS files were submitted to ProteinPilot (version 4.5,^1^ SCIEX, Redwood City, CA, USA) for analysis. ProteinPilot’s Paragon algorithm was used to search the UniProtKB-A1E5T4 (A1E5T4_FASGI) database (access time is 2021/11/19) and identify proteins using the following parameters: TripleTOF 5,600, cysteine modification with iodoacetamide, and biological modification as the ID focus. The identified protein results were subject to certain filtering criteria. Peptides with an unused score > 1.3 (credibility of >95%) were considered credible, and proteins containing at ≥1 unique peptide were retained.

## 3. Results

### 3.1. *Fasciola gigantica* infection confirmation

*Fasciola gigantica* infection was confirmed in the three buffaloes in the infection group based on positive indirect *Fg*ESP-based ELISA findings 2 wpi. *F. gigantica* eggs were also detected in the faeces between 12 and 14 wpi. In addition, autopsies at 16 wpi found livers from the infection group to show obvious gross pathological lesions, and adult flukes were detected and the mean numbers of flukes recovered were 55.5 ± 14.1 (22.2 ± 5.6 of infection dose), indicating established infections (Wang et al., 2022a). All buffaloes in the non-infection group had negative indirect *Fg*ESP-based ELISA findings.

### 3.2. SDS-PAGE confirmation

**Figure 1:**
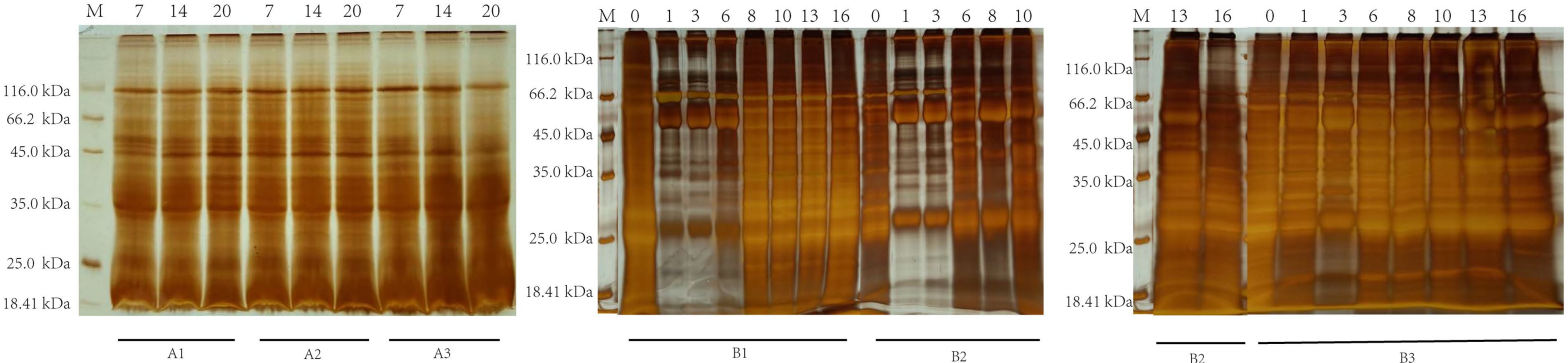
SDS-PAGE analysis of buffalo serum cocultured with *Fg*ESPs during different infection periods. The numerical value above represents the serum’s wpi. The capital letter below represents the ID of buffaloes from the non-infection (A1, A2, and A3) and infection (B1, B2, and B3) groups.

SDS-PAGE indicated that serum-derived antibodies could recognize and pull down specific *Fg*ESP components at different infection periods in the non-infection and infection groups (Figure 1). The molecular weights of majority of specific proteins identified and pulled down by non-infection groups ranged from 25.0 kDa to 116.0 kDa, while infection group ranged from 18.41 kDa to 116.0 kDa.

### 3.3. LC–MS/MS analysis of non-infection and infection groups

In the non-infection group, individual buffalo were applied to analyze the percent of interacting proteins in three stages of infection: early (3 wpi), middle (10 wpi) and late (16 wpi), and the effect of external environment on the experiment was evaluated by the percentage of the number of shared proteins identified in the three stages compared with the total number of proteins in the three stages. A1W3:318/395 = 80.5%; A1W10:318/426 = 74.6%; A1W16:318/416 = 76.4%, A2 and A3 were also displayed (Table 1).

**Table 1:** The percent of shared number accounting number of specific week in the non-infection group buffaloes.

	3&10&16 wpi	3 wpi		10 wpi		16 wpi	
	Shared number	Number (N3)	Percent (%) shared / N3	Number (N10)	Percent (%) shared / N10	Number (N16)	Percent (%) shared / N16
A1	318	395	80.5	426	74.6	416	76.4
A2	313	418	74.9	416	74.2	425	73.6
A3	299	355	84.2	413	72.4	409	73.1

**Figure 2:**
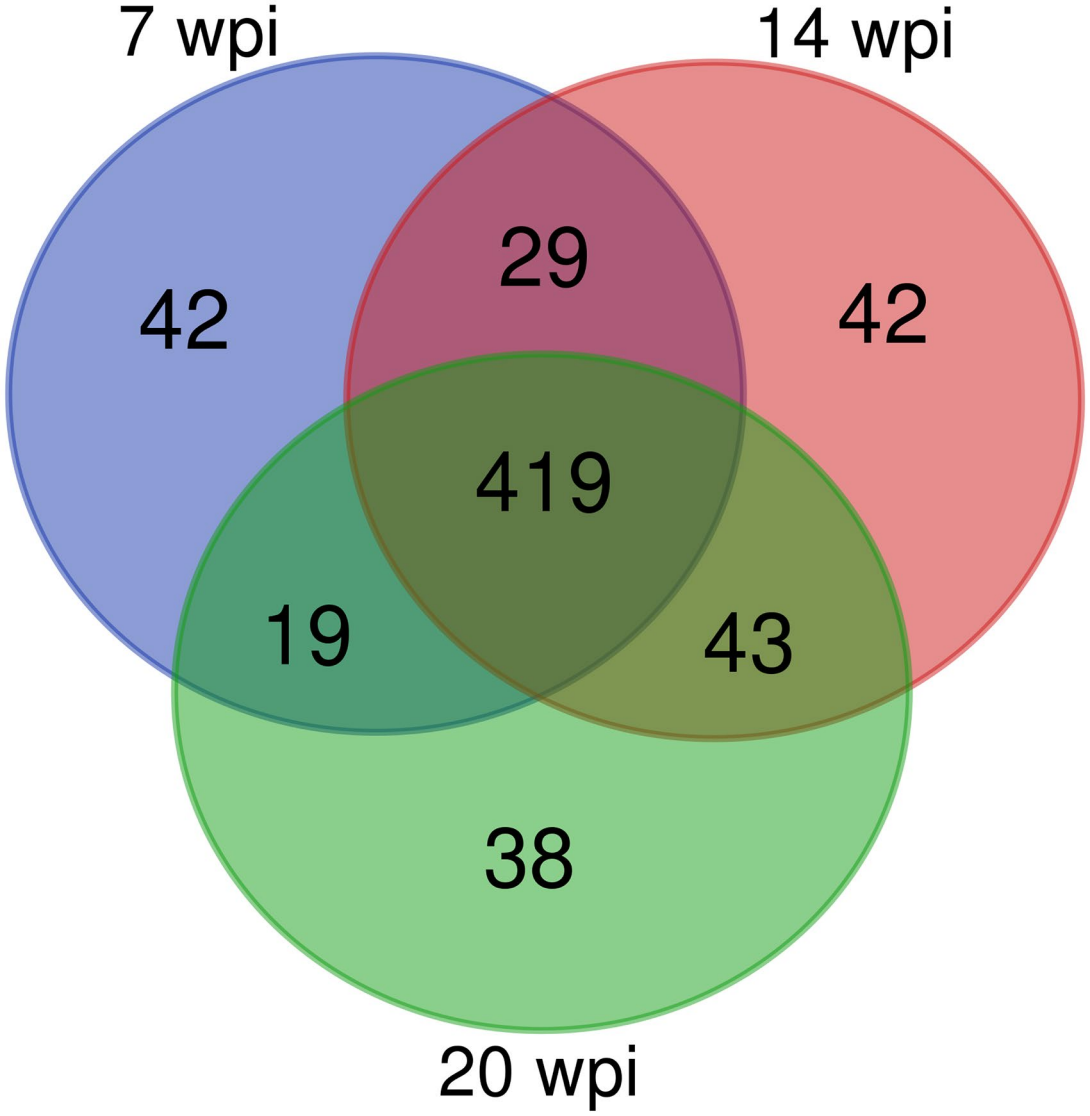
A Venn diagram showing the number of overlapping proteins pulled down by non-infection group’s buffalo serum using the *Fg*ESPs.

Overall, 509, 533, and 519 specific proteins were identified in buffaloes A1, A2, and A3, of which 419 were identified in all three, accounting for 82.3, 78.6, and 80.7% of all proteins identified at 3, 10, and 16 wpi, respectively (Table 2; Figure 2). As total of 632 proteins were identified in all three buffaloes, 3 wpi accounting for 80.5% of all proteins identified, 10 and 16 wpi accounting for 84.3, and 82.1% of all proteins identified, respectively (Table 2; Supplementary Table S3).

**Figure 3:**
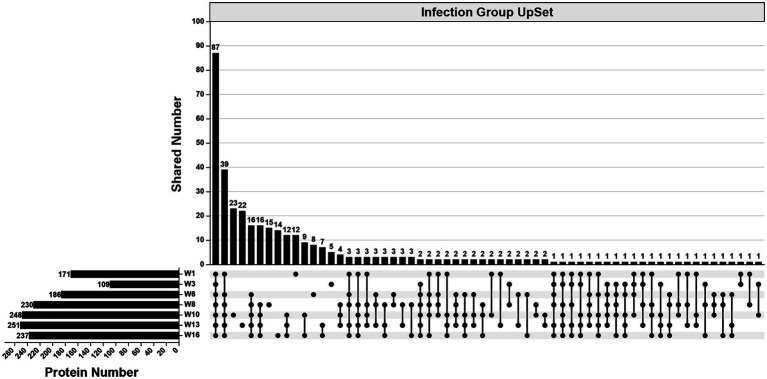
An UpSet diagram showing the number of unique and shared proteins in the infection group across 1, 3, 6, 8, 10, 13, and 16 wpi.

In the infection group, 490 specific proteins were identified across all examined wpi. The numbers identified were 171 (1 wpi), 109 (3 wpi), 186 (6 wpi), 230 (8 wpi), 248 (10 wpi), 251 (13 wpi), and 237 (16 wpi). Overall, 87 proteins were identified consistently across all examined wpi. The numbers of specific proteins to each wpi were 12 (1 wpi), 5 (3 wpi), 8 (6 wpi), 15 (8 wpi), 23 (10 wpi), 22 (13 wpi), and 14 (16 wpi), respectively (Figure 3).

**Table 2:** The percent of shared number accounting the number of specific week and the number of specific weeks accounting the number of all periods in the non-infection group.

wpi	Shared number	Number of specific week (N1)	Percent (%) shared / N1	Number of three periods (N2)	Percent (%) N1 / N2
3	419	509	82.3	632	80.5
10		533	78.6		84.3
16		519	80.7		82.1

### 3.4. Analysis of consistently detected proteins in the infection group

**Figure 4:**
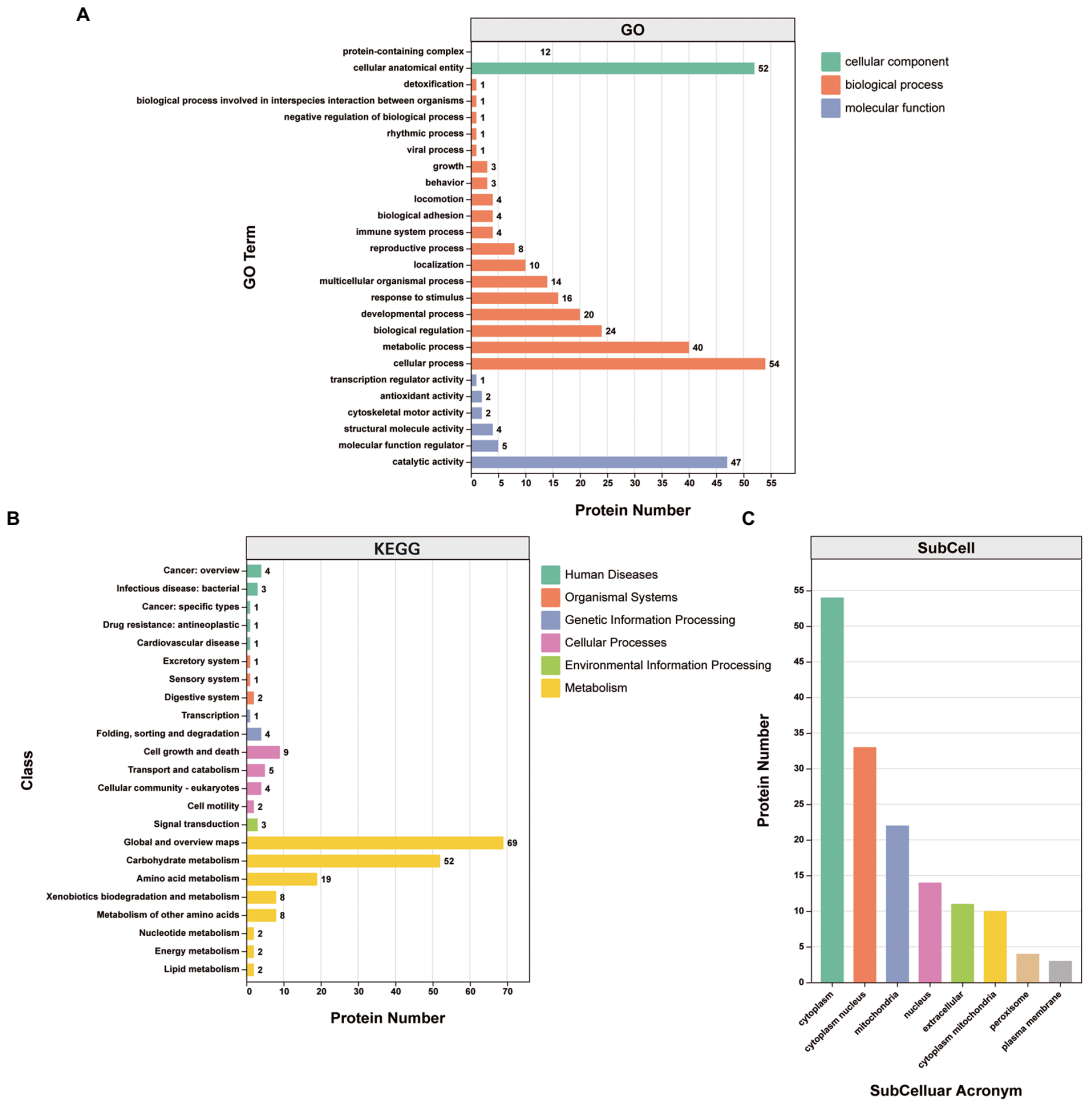
Analysis of the 87 proteins consistently identified in the infection group. **(A)** GO annotation clustered the proteins into three categories: molecular function, cellular component, and biological process. GO annotation and classifications are shown based on secondary names. While the horizontal axis represents protein numbers, the vertical axis represents GO secondary names. **(B)** KEGG pathway protein annotation and its corresponding category in the KEGG database are shown in different colors. While the horizontal axis represents protein numbers, the vertical axis represents the KEGG class names. **(C)** Protein subcellular localization. While the horizontal axis represents subcellular classification, the vertical axis represents protein numbers.

Gene Ontology (GO) classification was used to investigate the biological function of the 87 proteins consistently identified in the infection group. They were clustered into the “biological process,” “cellular component,” and “molecular function” categories. Within the “biological process” category, proteins clustered in the “cellular process” (25.8%), “metabolic process” (19.1%), “biological regulation” (11.5%), “developmental process” (9.6%), “response to stimulus” (7.7%), and “multicellular organismal process” (6.7%) subcategories. Within the “cellular component” category, the proteins clustered in the “cellular anatomical entity” (81.2%) and “protein-containing complex” (18.8%) subcategories. Within the “molecular function” category, the proteins mainly clustered in the “binding” (46.0%) and “catalytic activity” (41.6%) subcategories, with other subcategories accounting for much smaller proportions (Figure 4A; Supplementary Table S4).

Kyoto Encyclopedia of Genes and Genomes (KEGG) annotations suggested that the most abundant pathways represented by the 87 proteins were “global and overview maps,” “amino acid metabolism,” and “carbohydrate metabolism” in “metabolism,” followed by “cell growth and death” in “cellular processes” (Figure 4B; Table 3). Furthermore, 24 of the 87 proteins were annotated in more than one KEGG pathway (Supplementary Table S4). These included glycometabolism-related proteins, such as phosphoglucomutase, glutamate dehydrogenase, UTP-glucose-1-phosphate uridylyltransferase, fructose-bisphosphate aldolase, and malate dehydrogenase, which were annotated in ≥5 KEGG pathways. In addition, 14–3-3 proteins (Chaithirayanon et al., 2004; Tian et al., 2018), ferritin (Caban-Hernandez et al., 2012), Fh5 (Rossjohn et al., 1997), and heat shock proteins (HSPs; Moxon et al., 2010) were also annotated in KEGG pathways.

**Table 3:** Partial proteins consistently identified in the infection group.

Acc	Protein description	Peptide	Unique peptide	Coverage (%)	Length	Mass
tr|A0A504Z0W5|A0A504Z0W5_FASGI	Fh51	1	1	15.89	107	1,2544.3
tr|A0A504Z3A0|A0A504Z3A0_FASGI	Prostaglandin-H2 D-isomerase	14	14	66.82	211	2,4568.3
tr|A0A504YW63|A0A504YW63_FASGI	Glutathione transferase	10	10	29.07	313	3,5968.5
tr|A0A504YYH3|A0A504YYH3_FASGI	Cardiac muscle alpha actin	15	1	41.07	375	4,1696.3
tr|A0A504YG42|A0A504YG42_FASGI	Heat shock protein heat shock protein 90 alpha	27	25	40.3	722	8,2427.7
tr|A0A504YUP2|A0A504YUP2_FASGI	Phosphoenolpyruvate carboxykinase (GTP)	36	36	70	550	6,1415.2
tr|A0A504YN48|A0A504YN48_FASGI	cAMP-dependent protein kinase catalytic subunit alpha	3	3	14.02	321	37,344
tr|A0A504YX93|A0A504YX93_FASGI	Tyrosine 3-monooxygenase/tryptophan 5-monooxygenase activation protein theta polypeptide	15	13	66.27	252	2,8661.1

The subcellular localizations of the 87 proteins were cytoplasmic (35.8%), cytoplasm and nucleus (21.9%), and mitochondrial (14.6%; Figure 4C; Supplementary Table S4).

### 3.5. Specific proteins detected in the infection group

Partial proteins detected in *Fg*ESPs from the buffaloes’ sera single and multiple wpi are shown in Supplementary Table S5. Since this study has described or clustered these proteins into specific KEGG pathways, their functions, such as calcium binding, could be inferred. Complete lists of proteins identified at a single wpi or across multiple wpis are provided in Supplementary Tables S6, S7.

## 4. Discussion

This study used SDS-PAGE to confirm the co-IP assay. The non-infection control group showed that many identified proteins were shared across time points (3, 10, and 16 wpi), suggesting that the buffaloes’ surroundings did not affect their immune response during maintenance. While we identified numerous proteins in the infection group already reported with *F. gigantica*, we described some unique proteins associated with *F. gigantica* and used KEGG database and subcellular localization analyses to infer their potential functions.

KEGG analysis of the 87 proteins continuously identified in the infection group showed that some are associated with various signaling pathways (Table 3), including cytochrome-P450-related drug metabolism (Fh51, prostaglandin-H2 D-isomerase, and glutathione transferase), hippo signaling (cardiac muscle alpha-actin), estrogen signaling (HSP90 alpha [HSP90α]), interleukin (IL)-17 signaling (HSP90α), Th17 cell differentiation (HSP90α), phosphoinositide 3-kinase (PI3K)-protein kinase B (AKT) signaling (HSP90α), nucleotide-binding oligomeric domain (NOD)-like receptor (NLR) signaling (HSP90α), forkhead box O (FOXO) signaling (phosphoenolpyruvate carboxykinase), Wnt signaling (cAMP-dependent protein kinase catalytic subunit alpha), and longevity regulation (activation protein theta polypeptide).

Anthelmintics can be neutralized or bio-transformed jointly or independently by three protein-level defense systems, termed phases I to III (Cvilink et al., 2008). In vertebrates and most invertebrates, the phase I pathway is oxidative *via* the cytochrome P450 superfamily (Brophy et al., 2012). However, parasitic helminths are much less able to neutralize external toxins (xenobiotics) than their mammalian hosts (Cvilink et al., 2009), potentially reflecting their lack of important phase I cytochrome P-450-dependent detoxification components. Studies have shown that Glutathione-S-transferase (GST), ATP-binding cassette (ABC), fatty acid-binding protein and adenosine deaminase (ADA) in the excretory products of fluke functions in detoxification during the parasitic process (Morphew et al., 2007; Kumkate et al., 2008; Kalita et al., 2017; Rehman et al., 2020), and the alteration of ADA activity could induce the host immune responses switch to Th-2 type and facilitate the establishment of flukes within their host (Rehman et al., 2021). In addition to the above proteins, KEGG analysis in the present study showing that Fh51, prostaglandin-H2 D-isomerase, and glutathione transferase has been identified and clustered to cytochrome-P450-related xenobiotic (drug) metabolism, indicating these three proteins may also involve in detoxification during *F. gigantica* parasitism (Alirahmi et al., 2010; Kalita et al., 2019).

As a highly conserved molecular chaperone protein, HSP90 involved in signal transduction, cell cycle control, stress management and folding, degradation, and transport of proteins (Johnson, 2012; Roy et al., 2012; Gillan and Devaney, 2014; Hoter et al., 2018; Zininga et al., 2018; Biebl and Buchner, 2019; Backe et al., 2020). HSP90 also has been thought involved in host immune system modulation *via* platyhelminth secretomes (Liu et al., 2009; Xu et al., 2020). There are two cellular subtypes of HSP90, while HSP90α isoforms been secreted from cells, HSP90β isoforms (HSP90β) primarily operate intracellularly (Jayaprakash et al., 2015). In this study, HSP90α has been identified and clustered to interleukin (IL)-17 signaling, Th17 cell differentiation, phosphoinositide 3-kinase (PI3K)-protein kinase B (AKT) signaling, nucleotide-binding oligomeric domain (NOD)-like receptor (NLR) signaling.

The IL-17 family is a cytokine subgroup that plays crucial roles in host defense against microbes and inflammatory disease development (Chen et al., 2017). IL-17E (also called IL-25) is associated with type 2 T helper cell (Th2) response, promoting Th2-related cytokine production for eosinophil recruitment and contributing to host defense against parasitic helminth infections (Pan et al., 2001; Ballantyne et al., 2007; Saenz et al., 2008; Kang et al., 2012). Recently, researchers found that peripheral blood lymphocytes (pBLs) significant upregulated Th2/Th17 type immune response at 3 and 42 dpi in buffaloes infected with *F. gigantica* (Hu et al., 2022), which was consistent with previous studies showing that the Th1-related response is inhibited early in *F. gigantica* infection, while the Th2-related response favoring parasitism is promoted (Molina, 2005; Rodriguez et al., 2017). HSP90α may regulates the IL-17 signaling pathway during early infection, enabling *F. gigantica* host parasitism. Therefore, it can infer that *F. gigantica* further participates in the Th2 / Th17 type immune response by secreting HSP90α in the IL-17 signaling pathway in the early stage of infection, thus regulating the host immune developed to a direction conducive to fluke survival.

Although HSP90α was identified in *Fg*ESP, it may also function in intracellular process. The PI3K-AKT signaling pathway regulates the number of neoblast/pluripotent cells in *Schmidtea mediterranea* (Peiris et al., 2016) and is essential for enhancing pluripotent cell survival (Hossini et al., 2016). Neoblast/pluripotent cells were produced and proliferated throughout the *F. hepatica* life cycle (McCusker et al., 2016; Cwiklinski et al., 2018), suggesting its key role in *Fasciola* growth and development. Studies have identified cell surface location-chaperone, and assign their functions to the recognition of infectious agents or their components and subsequent intracellular signaling (Henderson et al., 2006). Considering molecular chaperone characteristic of HSP90α, together with its clustering to PI3K-AKT signaling pathway, HSP90α was supposed to regulate the Neoblast/pluripotent through the PI3K-AKT signaling pathway, which ultimately regulate the growth and development of *F. gigantica*. Cytoplasmic NLRs function as innate pattern recognition receptors, the first line of defense against microbial infection (Zhang et al., 2018) that recognize pathogens, recruit innate immune cells, and activate adaptive immune responses (Fukata et al., 2009; Cooney et al., 2010). NOD1 and NOD2 proteins can be recruited to the plasma membrane and regulate nuclear factor kappa-light chain enhancers of activated B-cell signaling and mitogen-activated protein kinase (MAPK) pathway (Philpott et al., 2014). We hypothesize that HSP90α suppresses host innate and adaptive immune responses through the NLR signaling pathway, enhancing *F. gigantica* survival.

Wnt signaling pathway including canonical Wnt β-catenin-dependent and non-canonical Wnt/Ca2+ signaling pathways (Ovchinnikov et al., 2020), which can initiate and regulate various cellular activities (including cell proliferation and calcium homeostasis), regulate the establishment of the anterior–posterior axis (AP axis) and the medial-lateral axis (Petersen and Reddien, 2009; De Robertis, 2010), also involved in the neural system formation (Adell et al., 2009). While canonical pathway can be modulated to alter glucose concentrations in the blood and surrounding tissues (Zhou et al., 2014; Chen et al., 2018), the non-canonical pathway mediates inflammatory responses, leading to suppression of host inflammatory responses by inhibiting positive feedback mechanisms (De, 2011).

Some of the 87 proteins consistently identified in the infection group were not assigned a KEGG signaling pathway, including ferritin. A recent study showed that ferritin in *Fh*ESPs separated by 2D electrophoresis did not react with infected sheep serum, suggesting that ferritin was a non-immunogenic *Fh*ESP protein (Becerro-Recio et al., 2021). However, this study found that ferritin consistently reacted with serum from *F. gigantica*-infected buffalo, indicating that ferritin in *Fg*ESPs is a complete antigen. Therefore, ferritin’s function in *Fg*ESPs needs to be explored further.

Five of the 19 proteins consistently identified during the invasive infection phase (1–3 wpi) were uncharacterized (Supplementary Tables S6, S7). The microtubule-associated protein Futsch was associated with biological processes in GO taxonomic annotation. Microtubulin is a benzimidazole (BZ) target extensively studied in parasitology (von Samson-Himmelstjerna et al., 2007). A study using triclabendazole (TCBZ), a BZ derivative used to treat fascioliasis, showed that *F. hepatica*’s microtubule-mediated functions were inhibited by TCBZ exposure, suggesting that microtubule proteins may be effective TCBZ targets (Hanna, 2015).

Polyubiquitin proteins and three histones (H2A, H2B, and H3) were identified at 1 wpi. After excystation, NEJs interact with intestinal epithelial cells and inhibit the immune cell signaling cascade by downregulating intracellular signaling and the downstream ubiquitination-associated proteins required to trigger the immune response (Lammas and Duffus, 1983; Dalton et al., 2009; Lalor et al., 2021). Molecules secreted or excreted during this stage (1–3 wpi) likely play vital roles in host invasion and have the potential to be candidate vaccine/drug targets to inhibit NEJ infestation and migration.

Four of the 26 proteins identified between 6 and 8 wpi were uncharacterized (Supplementary Tables S6, S7). Programmed cell death 6-interacting protein and Thimet oligopeptidase (M03 family) were identified at both 6 and 8 wpi. GO analysis of T-complex protein 1 subunit γ, annexin, and dynein beta chain ciliary protein, which were only identified at 6 wpi, identified their localization and motility functions. Constitutive HSP70, HSP90 chaperone protein kinase-targeting subunit, glycerol-3-phosphate dehydrogenase (nicotinamide adenine dinucleotide), succinate dehydrogenase (ubiquinone) iron–sulfur subunit, mitochondria (fragment), and puromycin-sensitive aminopeptidase were only identified at 8 wpi. KEGG analysis showed a functional focus on energy metabolism, including oxidative phosphorylation, the citric acid cycle, starch and sucrose metabolism, purine metabolism, pyrimidine metabolism, and nicotinic acid and nicotinamide metabolism. Between 6 and 8 wpi, *Fasciola* migrate to the host’s liver and induce high oxidative stress levels (Da Silva et al., 2017). HSP70 may function in protein folding and assembly, refolding misfolded and aggregated proteins, and transferring proteins to mediate the environmental stress and cellular homeostasis effects, which is critical for parasite survival (Polla, 1991; Mayer and Bukau, 2005; Smith et al., 2008). The active metabolic pathways provide the nutrients for *F. hepatica* growth and development between 6 to 8 wpi (Tanaka and Miyajima, 2016), and it may be similar in *F. gigantica* growth and development.

Long-term *F. gigantica* survival requires a balance between immuno-suppressive and-modulatory effects induced by *F. gigantica* and the host’s innate and adaptive immune responses. Twelve of the 78 proteins identified between 10 and 16 wpi were uncharacterized (Supplementary Tables S6, S7). Legumain-like calcium-binding protein 39 and transforming growth factor-β (TGF-β)-inducible protein ig-h3 (fragment) were consistently identified during this period. KEGG pathway analysis indicated that they primarily function in metabolic pathways. Studies have shown that recombinant legumain is specifically recognized by positive sera from *F. hepatica*-infected sheep, showing good reactogenicity (Zhang et al., 2021). Subsequent studies showed it differed biologically between *Schistosoma haematobium* and *F. gigantica*, indicating its vaccine potential against *F. gigantica* (Adisakwattana et al., 2007).

Tegumental calcium-binding EF-hand protein 4 (CABP4) was identified at both 10 and 13 wpi (Supplementary Table S6). The EF-hand is an important functional protein domain in *F. gigantica* calcium-binding protein (Santiago et al., 1998). The EF-hand-containing protein CABP4 is an important *Fg*ESP component that shows an immunomodulatory effect during *F. gigantica* infection (Subpipattana et al., 2012; Huang et al., 2019b; Ehsan et al., 2021). Studies investigating *Fh*CABP1, *Fh*CABP2, and *Fh*CaBP4 have been performed (Banford et al., 2013; Thomas and Timson, 2015; Cheung et al., 2016). However, relevant studies on *F. gigantica* calcium-binding proteins are lacking. Given the calcium-binding protein family’s ability to induce immunoglobulin E-mediated host immune responses (Santiago et al., 1998), there is a need to study their immunomodulatory functions in *F. gigantica*.

*Fasciola* migration in the liver triggers a wound-healing response that induces fibrosis to repair the damage (Dorey et al., 2021), culminating in liver fibrosis and granulomas. This progress may be related to forkhead box P3 (FOXP3)^+^ T regulatory cell (Treg) levels (Pacheco et al., 2018), regulatory cytokines (IL-10 and TGF-β), and proinflammatory cytokines (tumor necrosis factor-alpha and IL-1β; Valero et al., 2017). Here, the TGF-β-inducible protein ig-h3 (fragment) identified between 10 and 16 wpi may participate in the host tissue damage repair (Supplementary Table S5). The aldolase (fructose-bisphosphate) identified between 10 and 16 wpi is secreted by or attached to the epidermis of *Fasciola* (Morales and Espino, 2012). It mainly acts as a ligand for various host components contributing to fluke invasion (Zhang et al., 2019), host immune and hemostatic systems regulation, angiogenesis, and nutrient absorption (Gómez-Arreaza et al., 2014).

*Fg*ESPs are exposed to the host immune system and widely used as antigens in serological assays. Five of the 19 proteins identified between 1 and 3 wpi were uncharacterized (Supplementary Tables S6, S7). The specificity and sensibility of these proteins still need to be confirmed by Western blot and ELISA. Once some have been purified and shown to react well with positive serum, they can be used to develop new early-diagnosis antigen immunological diagnostic methods. However, this study did not identify well-performing early diagnosis antigens, such as cathepsin L and secreted aspartyl proteinase 2, indicating more accurate approaches may be needed to understand the precise buffalo-*F. gigantica* interaction (Cornelissen et al., 2001; Sriveny et al., 2006; Kueakhai et al., 2011; Mirzadeh et al., 2017).

During early infection stages, *F. gigantica* induces the Th2-related response and suppresses the Th1-related response in the host. Molecules functioning in this process are potential vaccine candidates (Donnelly et al., 2008; Walsh et al., 2009). Fifteen of the 96 proteins identified between 6 and 10 wpi were uncharacterized (Supplementary Tables S6, S7), including cathepsin L. Cathepsin-L peptidases have been extensively studied since they are internalized by host immune cells and degrade the pathogen recognition receptor Toll-like receptor 3, preventing Toll/IL-1R domain-containing adaptor-inducing interferon-β-containing adaptor protein-dependent signaling that is essential for the Th1 inflammatory response (Falcón et al., 2014). The mammalian target of rapamycin (mTOR), MAPK, and FOXO signaling pathways act synergistically to promote *FOXP3* expression and differentiation into Treg cells (Delgoffe et al., 2009). Treg cells secrete the regulatory cytokines TGF-β and IL-10, regulating the Th1-and Th2-related responses (Hill et al., 2007). This process may be related to immunomodulation and long-term host colonization (Maizels and Lawrence, 1991; King et al., 1992). Since calcium-binding protein 39, F-actin-capping protein subunit beta, and V-type proton ATPase subunit H clustered with the mTOR signaling pathway; constitutive HSP70 clustered with the MAPK signaling pathway; and the glucose transporter clustered with the FOXO signaling pathway, these proteins may regulate the Th1-and Th2-related responses. Studying their immunomodulatory functions may contribute to vaccine candidate identification.

## 5. Conclusion

This study performed a detailed screening of antigenic *Fg*ESP targets, as 490 proteins were identified in the infection group, of which 87 were consistently identified at 7 time points, the numbers of specific interactors identified for each week were 1 (*n* = 12), 3 (*n* = 5), 6 (*n* = 8), 8 (*n* = 15), 10 (*n* = 23), 13 (*n* = 22), and 16 (*n* = 14) wpi. These findings will lay the foundation for further studies on *F. gigantica-*host interactions and fascioliasis diagnosis and prevention.

## Data availability statement

The data presented in the study are deposited in the ProteomeXchange repository, accession number PXD038582.

## Ethics statement

This animal study, including sera collection, was approved by the Ethics Committee of the School of Animal Science and Technology at Guangxi University. The animals used in this study were handled according to good animal practices as required by the Animal Ethics Procedures and Guidelines of the People’s Republic of China.

## Author contributions

WZ conceived the project. MZ performed the laboratory work and data analysis and wrote the manuscript. XJ, XK, and YG performed supporting data analyses. WD revised the manuscript and contributed to the final submission. All authors contributed to the article and approved the submitted version.

## Funding

This study was supported by the National Natural Science Foundation of China (Grant no. 31960706).

## Conflict of interest

The authors declare that the research was conducted in the absence of any commercial or financial relationships that could be construed as a potential conflict of interest.

## Publisher’s note

All claims expressed in this article are solely those of the authors and do not necessarily represent those of their affiliated organizations, or those of the publisher, the editors and the reviewers. Any product that may be evaluated in this article, or claim that may be made by its manufacturer, is not guaranteed or endorsed by the publisher.

## Footnotes

^1^ https://sciex.com.cn/products/software/
