# Supplementary material for: Proteomic analysis of Fasciola gigantica excretory and secretory products (FgESPs) co-immunoprecipitated using a time course of infected buffalo sera
Source: Front Microbiol. 2022 Dec 23;13:1089394. doi: 10.3389/fmicb.2022.1089394 (PMC9816151; doi:10.3389/fmicb.2022.1089394)
Supplement: Supplementary file 1 [file Table_1.DOCX]

Table S1. Basic information about experimental animals

| Buffaloes | Subspecies | Gender | Weight (Kg) |
| --- | --- | --- | --- |
| A1 | Murrah | Female | 306 |
| A2 | Murrah | Female | 288 |
| A3 | Murrah | Male | 317 |
| B1 | Murrah | Male | 288 |
| B2 | Hybrid-Mediterranean | Female | 320 |
| B3 | Hybrid-Nili-Ravi | Male | 380 |
