# Supplementary material for: Proteomic analysis of Fasciola gigantica excretory and secretory products (FgESPs) co-immunoprecipitated using a time course of infected buffalo sera
Source: Front Microbiol. 2022 Dec 23;13:1089394. doi: 10.3389/fmicb.2022.1089394 (PMC9816151; doi:10.3389/fmicb.2022.1089394)
Supplement: Supplementary file 2 [file Table_2.DOCX]

Table S2. The percentage of antibodies to the IgG of the experimental animal before infection

|  | Critical Value | Detection Time | | | | |
| --- | --- | --- | --- | --- | --- | --- |
|  |  | 2020/09/16 | 2020/10/13 | 2020/10/21 | 2020/11/05 | 2020/11/20 |
| A1 |  | 27.9% | 28.4% | 20.7% | 13.0% | 7.4% |
| A2 |  | -23.3% | -5.9% | -1.3% | 3.5% | -0.3% |
| A3 | 30% | 27.6% | 28.0% | 29.2% | 8.4% | -7.0% |
| B1 |  | 16.2% | 1.6% | 12.0% | 9.8% | 0.2% |
| B2 |  | -13.2% | -11.5% | -16.5% | -4.1% | -16.9% |
| B3 |  | 2.9% | 5.6% | 1.9% | 7.3% | 10.5% |
