# Supplementary material for: Proteomic analysis of Fasciola gigantica excretory and secretory products (FgESPs) co-immunoprecipitated using a time course of infected buffalo sera
Source: Front Microbiol. 2022 Dec 23;13:1089394. doi: 10.3389/fmicb.2022.1089394 (PMC9816151; doi:10.3389/fmicb.2022.1089394)
Supplement: Supplementary file 5 [file Table_5.docx]

Table S5. Partial specific proteins identified at single- and multi-wpi in the infection group.

| **Period**  **(wpi)** | **Acc** | **Protein Description** | **Peptide** | **Unique Peptide** | **Coverage**  **(%)** | **Length** | **Mass** |
| --- | --- | --- | --- | --- | --- | --- | --- |
| 1&3 | tr\|A0A504YJP1\|A0A504YJP1_FASGI | Microtubule-associated protein futsch | 1 | 1 | 1.033 | 968 | 103320 |
| 1 | tr\|A0A504YHV3\|A0A504YHV3_FASGI | Polyubiquitin | 2 | 2 | 22.92 | 192 | 21751.8 |
| 1 | tr\|A0A504YHG3\|A0A504YHG3_FASGI | Histone H2A | 2 | 2 | 22.4 | 125 | 13474.7 |
| 1 | tr\|A0A504Z6J7\|A0A504Z6J7_FASGI | Histone H2B | 2 | 2 | 16.39 | 122 | 13676.9 |
| 1 | tr\|A0A504YJX2\|A0A504YJX2_FASGI | Histone H3 | 1 | 1 | 4.186 | 215 | 24017.2 |
| 6&8 | tr\|A0A504YU64\|A0A504YU64_FASGI | Programmed cell death 6-interacting protein | 1 | 1 | 6.542 | 214 | 23686.8 |
| 6 | tr\|A0A504YS00\|A0A504YS00_FASGI | T-complex protein 1 subunit gamma | 1 | 1 | 2.015 | 546 | 59636.7 |
| 6 | tr\|A0A504YP52\|A0A504YP52_FASGI | Annexin | 1 | 1 | 1.754 | 912 | 101351.9 |
| 6 | tr\|A0A504YLU8\|A0A504YLU8_FASGI | Dynein beta chain ciliary | 1 | 1 | 0.1996 | 4008 | 457380.5 |
| 8 | tr\|A0A504Z2F6\|A0A504Z2F6_FASGI | Hsp90 chaperone protein kinase-targeting subunit | 2 | 2 | 4.489 | 401 | 46666.5 |
| 8 | tr\|A0A504Z3X5\|A0A504Z3X5_FASGI | Constitutive heat shock protein 70 | 8 | 1 | 11.37 | 651 | 71132.7 |
| 8 | tr\|A0A504YLE5\|A0A504YLE5_FASGI | Glycerol-3-phosphate dehydrogenase [NAD(+)] | 1 | 1 | 5.698 | 351 | 38696.5 |
| 8 | tr\|A0A504YRX6\|A0A504YRX6_FASGI | Succinate dehydrogenase [ubiquinone] iron-sulfur subunit, mitochondrial (Fragment) | 2 | 2 | 9.167 | 240 | 27838.2 |
| 8 | tr\|A0A504Y7V0\|A0A504Y7V0_FASGI | Puromycin-sensitive aminopeptidase | 1 | 1 | 3.571 | 252 | 28395.8 |
| 10&13&16 | tr\|A0A504YCT9\|A0A504YCT9_FASGI | Legumain like | 2 | 2 | 5.923 | 439 | 49585.9 |
| 10&13&16 | tr\|A0A504YXL7\|A0A504YXL7_FASGI | Calcium binding protein 39 | 2 | 2 | 6.158 | 406 | 46613 |
| 10&13&16 | tr\|A0A504YAW2\|A0A504YAW2_FASGI | Transforming growth factor-beta-induced protein ig-h3 (Fragment) | 1 | 1 | 2.491 | 562 | 64182.2 |
